# Sexual dysfunction and sexual quality of life among the physically challenged in the Kumasi metropolis, Ghana

**DOI:** 10.1186/s12955-015-0206-8

**Published:** 2015-01-22

**Authors:** William K B A Owiredu, Alexander O Owusu, Nafiu Amidu, Lawrence Quaye, Christian K Gyasi-Sarpong, Peter P M Dapare, Huseini Alidu

**Affiliations:** Department of Molecular Medicine, School of Medical Sciences, College of Health Sciences, Kwame Nkrumah University of Science and Technology, Kumasi, Ghana; Department of Biomedical Laboratory Science, School of Medicine and Health Sciences, University for Development Studies, Tamale, Ghana; Department of Surgery, (Urology Unit) Komfo Anokye Teaching Hospital/College of Health Sciences, Kwame Nkrumah University of Science and Technology, Kumasi, Ghana

## Abstract

**Background:**

Despite the fact that the physically disabled have difficulties in many aspects of their lives, including sexuality, society often ignores these needs or assume that they have no such needs. This cross-sectional study therefore seeks to determine the prevalence of sexual dysfunction (SD) and its impact on the quality of life among persons with physical disability residing in the Kumasi metropolis, Ghana.

**Method:**

This study was conducted among 235 persons with physical disability dwelling in communities within the Kumasi metropolis, Ghana between September 2011 and April 2012. All participants were evaluated by using a semi-structured questionnaire, the Golombok Rust Inventory of Sexual Satisfaction (GRISS) questionnaire and the Sexual Quality of Life questionnaire (SQoL). Self-designed semi-structured questionnaire was also administered to each consented study participant for socio-demographic information.

**Results:**

The response rates were 72% and 63.6% for male and female respectively. The age range of the male was 19–74 years with 61.1% being married whilst the age range of the female was 20–66 years with 54.3% being married. 30% and 7.1% of the male and female respectively consumed alcohol beverage. The mean Sexual quality of life (SQoL) score was slightly higher in the females (57.7 ± 15.8), ranging from 25.6 to 97.8. Univariate analysis of the male data showed that the only significant factor that tends to increase the male SD was alcohol (OR: 24.6; CI: 1.4 - 14.9; p = 0.0071). The prevalence of SD was higher among the female populace (65.7%) compared to the 64.4% for the male populace though very closely comparable. Except for non-communication (NC) and anorgasmia (impotence in males), all other areas of difficulty had higher percentages in males than females.

**Conclusion:**

The prevalence of sexual dysfunction among the physically challenged is comparable to prevalence rates in the able male and female population. This could impact significantly on their self-esteem and quality of life via avoidance, impotence and vaginismus thereby causing emotional distress leading to relationship problems. Alcohol increases the risk of developing SD by five-fold in physically challenged men.

## Background

Physical disability (PD) is a term used to describe any disorder which hinders or prevents the human body’s capabilities by altering its shape as well as structure [1]. PD could broadly be congenital or acquired, it may occur at all stages of life and helps in the quantification of the influence of disease or injury. Despite the fact that people with PD have challenges with many aspects of life, they do have natural needs like their able-bodied counterparts including sexual as well as emotional needs [2], although these may be with limitations depending on the nature and severity of their individual disability. Unfortunately, the sexual needs of people living with PD are not only unidentified and overlooked by the society; it is also assumed that they have no such needs.

Even though there is virtually no data on the prevalence of sexual function among people with PD, the rate of sexual functioning in the world database varies between 17.8% to 94.6%, depending on racial background, geographical location, socio-demographic status and environmental factors [3-8]. Among the able Ghanaian population, the prevalence of SD varies between 59.8% and 70.0% depending on the population [9,10], medical conditions [11,12] and marital status [13]. Among Ghanaian subjects with various medical conditions, the SD prevalence for subjects with self-reported diabetes was 70.0%, 50.0% among hypertensives, 41.7% among patients with migraine, 100.0% among ulcer patients, 75.0% among patients who have undergone surgery and 50.0% among STD patients [11,12]. Amidu *et al.* also recently reported the SD prevalence of 71.6% among Ghanaian men with urological conditions [14].

Persons living with PD may have permanent feelings of frustration and anxiety [2], which may worsen or initiate SD. A recent study has linked PD with reduced sexual satisfaction in persons with spinal cord injury [15]. SD compromises the overall quality of life of the individual and their sexual partners [16,17]. Gill *et al.* TM Gill, MM Desai, EA Gahbauer, TR Holford and CS Williams [18] have reported the powerful effects of disability on an individual’s well-being. To date, data are very scanty for developing countries including Ghana on the prevalence of SD among persons with PD. Available and relevant information on SD in Ghana are from the able-bodied population, hence the need to replicate such studies among this population. The objective of this cross-sectional study was to determine the prevalence of sexual dysfunction (SD) and its impact on the quality of life among persons with physical disability residing in the Kumasi metropolis, Ghana. To our knowledge, this is the first study of SD conducted among this group of individuals in Ghana.

## Methods

### Study participants

This cross-sectional study was conducted among 235 persons with physical disability dwelling in communities within the Kumasi metropolis, Ghana between September 2011 and April 2012. Eligibility criteria for participants were as follows, all sexually active subjects with physical disability, both male and female who are at least 18 years of age and are of sound mind. Participation of the respondents was voluntary and informed consent was obtained from each participant. Ethical consideration for this study was sought from the Committee on Human Research Publication and Ethics of the School of Medical Science and the Komfo Anokye Teaching Hospital, Kumasi (CHRPE).

### Sample size

The target group of this study was physically challenged men and women who are at least 18 years of age and are of sound mind within the Kumasi Metropolis. The necessary minimum sample size for the study was calculated to be 196 adults, based on the assumption that 85% of the physically challenged population experience sexual dysfunction [19], with an expected difference of 5% between the sample and the general population and a type I error (α) of 0.05.

$$ n=\frac{z^2\left(1-p\right)p}{d^2} $$

Where n = minimum sample size; Z = standard normal variance = 1.96 to obtain a power of 95% confidence interval (β = 5%) and a type 1 error probability of 5%; d = Absolute standard error = 0.05; p = prevalence = 85%.

In the present study, which was limited to only adult physically challenged men and women who answered all the evaluable questions in the questionnaire, the sample size was recalculated to evaluate any possible loss of precision. Given a targeted response rate of 90%, the sample size was recalculated as: 196/0.90. Using the above formula, the calculated sample size was approximately 218. Two hundred and thirty five (235) subjects were, therefore, recruited.

### Data collection

All participants were evaluated by using a semi-structured questionnaire, the Golombok Rust Inventory of Sexual Satisfaction (GRISS) questionnaire and the Sexual Quality of Life questionnaire (SQoL).

### Socio-demographic information

A detailed self-designed semi-structured questionnaire was administered to each consented study participant for socio-demographic information including age, marital status, behavioural activities (exercise, smoking and alcohol consumption), educational background, income level, type of disability and occupation. Exercise was defined as any activity causing light perspiration or a slight to moderate increase in breathing or heart rate for at least 30 minutes. Alcohol intake was defined as the intake of at least one bottle of an alcoholic beverage per week. Regarding smoking, individuals were classified as smokers based on whether the respondent is in the habit of smoking at least one cigarette a day.

### Golombok Rust Inventory of Sexual Satisfaction (GRISS)

Sexual response was measured by the Golombok Rust Inventory of Sexual Satisfaction (GRISS) questionnaire. The GRISS, in separate forms for men and women, has 28 items on a single sheet and is used for assessing the existence and severity of sexual problems in heterosexual couples or individuals who have a current heterosexual relationship. All the 28 questions are answered on a five-point (Likert type) scale from “always”, through “usually”, “sometimes”, and “hardly ever”, to “never”. It provides overall scores of the quality of sexual functioning within a relationship for men and women separately. In addition, subscale scores of impotence, premature ejaculation, anorgasmia, vaginismus, infrequency, non-communication, male dissatisfaction, female dissatisfaction, male non-sensuality, female non-sensuality, male avoidance and female avoidance can be obtained and represented as a profile. Responses are summed up to give a total raw score (range 28–140). The total score and subscale scores are transformed using a standard nine point scale, with high scores indicating greater problems. Scores of five or more are considered to indicate SD [20]. The GRISS was chosen because it is standardized, easy to administer and score, relatively unobtrusive and substantially inexpensive.

The GRISS can be used to assess improvement as a result of sexual or marital therapy and to compare the efficacy of different treatment methods. It can also be used to investigate the relationship between sexual dysfunction and extraneous variables. The subscales are particularly helpful in providing a profile for diagnosis of the pattern of sexual functioning within the couple, which can be of great benefit in designing a treatment program. The reliability of the overall scales has been found to be 0.94 for men, 0.87 for women and that of the subscales on average 0.74 (ranging between 0.61 and 0.83). Validity has been demonstrated under a variety of circumstances [20-22].

### Sexual quality of life (SQoL)

The sexual quality of life-male (SQoL-M) contains 11 items whilst SQoL-Female has 18 items each with a 6-point Likert-like response scale ranging from *‘completely agree’* to *‘completely disagree’*. The participants were asked to rate each item according to how much they agree or disagree with the statement by circling one of six categories. Items were scored 1–6 (worst to best) and were scored from *Completely Agree* = 1 to *Completely Disagree* = 6. In answering these items the following definitions apply: Sexual life - is both the physical sexual activities and the emotional sexual relationship that the individual have with their partner. Sexual activity - includes any activity which may result in sexual stimulation or sexual pleasure e.g. intercourse, caressing, foreplay, masturbation (i.e. self-masturbation or partner masturbation) and oral sex. To allow easy comparisons with other measures, raw scores were transformed onto a standardized scale of 0 to 100.

### Statistical analysis

All analyses were performed using GraphPad version 5.0, San Diego California, USA. The data was presented as mean ± SD or percentages. Logistic regression was used to assess the influence of different variables on sexuality. In all the statistical analysis, a value of p < 0.05 was considered significant.

## Results

### Response rate and socio-demographic characteristic

In all, 125 questionnaires were administered to the males, of which 122 (97.6%) were returned. The questionnaires from 32 men were rejected since they were incomplete, leaving 90 complete and evaluable questionnaires, indicating a response rate of 72%. For the females, out of a total of 110 questionnaires administered, 86 (78.2%) returned theirs. Out of these, 16 questionnaires were rejected as they were incomplete, indicating a response rate of 63.6%.

For the males, the age range for those who responded was between 19 to 74 years, with a mean age ± standard deviation 40.8 ± 12.5 whereas the female respondents ranged from age 20 to 66 years. For both males and female respondents, as shown in Table 1, majority were married, with percentages of 61.1% and 54.3% for males and females respectively. Basic education was the highest level of education attained by a majority of both male and female respondents; though the percentage of females in this bracket was more (60.0%) than the men (42.2%) whilst the other educational levels comprised less than 30% for both males and females. None of the respondents, both males and females were smokers whilst the males against females who took in alcohol were 30% against 7.1% respectively. Most of the males (67.8%) underwent some form of physical exercise each week compared to the 47.1% of females who did same. The income levels for the males ranged from GHc 10.00 to GHc 1400 per month with a mean ± standard deviation of 184.1 ± 223.2 whereas for the females, it ranged from GH c 10.00 to GH c 800 per month, with a mean ± standard deviation of 125.9 ± 165.8. Majority of both male and female respondents reported an acquired type of disability; that for the males being 67.8% and 72.9% for females. The inherited form of disability was on the low side among both males and females, with an overall percentage of almost 30%.

**Table 1:** **Socio-demographic characteristics of the study population**

**Variables**	**Total (n = 160)**	**Male (n = 90)**	**Female (n = 70)**	**P- value**
***Age (yrs)***	39.9 ± 11.8	40.8 ± 12.5	38.7 ± 10.8	0.2685
***Marital status***				
Married	93 (58.1%)	55 (61.1%)	38 (54.3%)	0.4217
***Educational level***				
Basic	80 (50.0%)	38 (42.2%)	42 (60.0%)	0.0379
Secondary	31 (19.4%)	17 (18.9%)	14 (20.0%)	1.0000
Technical	12 (7.5%)	9 (10.0%)	3 (4.3%)	0.2317
Tertiary	37 (23.1%)	26 (28.9%)	11 (15.7%)	0.0596
***Non-smokers***	160 (100.0%)	90 (100.0%)	70 (100.0%)	
***Alcohol consumption***	32 (20.0%)	27 (30.0%)	5 (7.1%)	0.0003
***Exercise***	94 (58.8%)	61 (67.8%)	33 (47.1%)	0.0099
***Income level (Ghc)***	159.7 ± 202.6	184.1 ± 223.2	125.9 ± 165.8	0.0780
***Type of disability***				
Acquired	112 (70.0%)	61 (67.8%)	51 (72.9%)	0.6022
Congenital	48 (30.0%)	29 (32.2%)	19 (27.1%)	0.6022

The mean raw scores and stanine scores for both male and female respondents is presented in Table 2. The male sexual dysfunction (SD) raw score ranged from 57 to 125 with a mean of 83.1 ± 12.8, whereas the female SD raw score was between 47 and 107, with a mean of 79.5 ± 11.5. The mean stanine scores for SD as well as all other subscales for both males and females were between 4.8 and 5.1 with a standard deviation ranging from 1.9 to 2.1. The SQoL score for males ranged from 12.1 to 83.3, with a mean of 45.7 ± 22.9. The mean SQoL score was slightly higher in the females (57.7 ± 15.8), ranging from 25.6 to 97.8.

**Table 2:** **GRISS raw scores and stanine scores for various subscales of the study population**

**Variables**	**Total (n = 160)**	**Male (n = 90)**	**Female (n = 70)**	**P- value**
**GRISS raw score**				
Sexual dysfunction	81.5 ± 12.3	83.1 ± 12.8	79.5 ± 11.5	0.0716
Avoidance	10.7 ± 3.3	10.7 ± 3.0	10.7 ± 3.7	0.9177
Non-sensuality	11.2 ± 3.2	11.1 ± 3.0	11.3 ± 3.4	0.6415
Infrequency	6.2 ± 1.8	6.4 ± 1.8	6.0 ± 1.9	0.1895
Impotence		12.1 ± 2.4		
Pre-mature ejaculation		11.5 ± 3.2		
Vaginismus			10.8 ± 3.6	
Anorgasmia			11.9 ± 3.1	
Non-communication	5.8 ± 1.9	5.6 ± 1.7	6.0 ± 2.2	0.2143
Dissatisfaction	12.6 ± 5.0	12.8 ± 6.1	12.4 ± 3.2	0.6004
**Stanine score**				
Sexual dysfunction	5.0 ± 1.9	5.0 ± 1.9	5.0 ± 1.9	0.9425
Avoidance	5.0 ± 2.0	5.1 ± 2.0	4.9 ± 2.0	0.4411
Non-sensuality	5.0 ± 2.1	5.0 ± 1.9	5.0 ± 2.0	0.7805
Infrequency	5.0 ± 2.0	5.0 ± 2.2	5.0 ± 1.9	0.8494
Impotence		5.0 ± 2.1		
Pre-mature ejaculation		5.1 ± 2.0		
Vaginismus			5.1 ± 2.0	
Anorgasmia			4.9 ± 2.0	
Non-communication	4.9 ± 2.1	4.8 ± 2.0	5.0 ± 2.2	0.5783
Dissatisfaction	4.9 ± 2.0	5.0 ± 2.0	4.9 ± 2.1	0.8723
**Quality of life**				
SQoL	50.9 ± 20.9	45.7 ± 22.9	57.7 ± 15.8	0.0003

### Risk factors

The effect of different socio-demographic variables on the risk of SD for both males and females are recorded in Tables 3 and 4 respectively. Univariate analysis of the male data showed that the only significant factor that tends to increase the male SD was alcohol (OR: 24.6; CI: 1.4 - 14.9; p = 0.0071); none of the other factors modified the SD of the males significantly. Similar analysis of the female data showed no associated risk factors with the ability to modify the SD significantly.

**Table 3:** **Univariate analysis of risk factors for male sexual dysfunction**

**Variables**	n/N*	**Rate of SD (%)**	**OR (CI 95%)**	**P value**
***Exercise***				
Yes	37/61	60.7	0.6 (0.2 - 1.5)	0.2761
No	21/29	72.4		
***Alcohol***				
Yes	23/27	85.2	4.6 (1.4 - 14.9)	0.0071
No	35/63	55.6		
***Married***				
Yes	37/56	66.1	1.2 (0.5 - 2.9)	0.6790
No	21/34	61.8		
***Educational attainment***				
Basic	30/38	78.9		
Secondary	11/17	64.7	0.5 (0.1 - 1.7)	0.2625
Technical	5/9	55.6	0.3 (0.1 - 1.5)	0.1479
Tertiary	12/26	46.2	0.2 (0.1 - 0.7)	0.0067
***Age ≥ 36 years***				
Yes	37/56	66.1	1.2 (0.5 - 2.9)	0.6790
No	21/34	61.8		
***Type of disability***				
Acquired	40/61	65.6	1.2 (0.5 - 2.9)	0.7455
Congenital	18/29	62.1		

*Number of subjects with SD/number of subjects in each category.

**Table 4:** **Univariate analysis of risk factors for female sexual dysfunction**

	n/N*	**Rate of SD (%)**	**OR (CI 95%)**	**P value**
***Exercise***				
Yes	21/33	63.6	0.8 (0.3 - 2.3)	0.7294
No	25/37	67.6		
***Alcohol***				
Yes	2/5	40	0.3 (0.0 - 2.1)	0.2087
No	44/65	67.7		
***Married***				
Yes	25/39	64.1	0.9 (0.3 - 2.3)	0.7500
No	21/31	67.7		
***Educational attainment***				
Basic	26/42	61.9		
Secondary	11/14	78.6	2.3 (0.5 - 9.3)	0.2540
Technical	2/3	66.7	1.2 (0.1 - 14.7)	0.8695
Tertiary	7/11	63.6	1.1 (0.3 - 4.3)	0.9160
***Age ≥ 36 years***				
Yes	21/39	64.1	0.6 (0.2 - 1.5)	0.2385
No	21/31	67.7		
***Type of disability***				
Acquired	33/51	64.7	0.8 (0.3 - 2.6)	0.7709
Congenital	13/19	68.4		

*Number of subjects with SD/number of subjects in each category.

### Sexual function-GRISS

The prevalence of SD was higher among the female populace (65.7%) compared to the 64.4% for the male populace though very closely comparable. Except for non-communication (NC) and anorgasmia (impotence in males), all other areas of difficulty had higher percentages in males than females. For the males the most prevalent areas of difficulty were infrequency (67.8%) followed by premature ejaculation (63.3%) and then non-sensuality (62.2%). The least prevalent areas of difficulty in the males were non-communication (55.6%) followed by dissatisfaction (60.0%). For the most prevalent areas of severe difficulties, infrequency still enjoyed a high percentage (14.4%). Non-communication however also came out as a highly prevalent area of severe difficulty (14.4%) despite recording the lowest prevalent area of difficulty. The other most prevalent areas of severe difficulty included premature ejaculation again (12.2%) and non-sensuality (11.1%). The least prevalent area of severe difficulty was dissatisfaction (1.1%). In the female populace, the most prevalent areas of difficulty were anorgasmia (67.1%), followed by non-communication (61.4%). Other prevalent areas of difficulty included avoidance (58.6%), vaginismus (58.6%) and dissatisfaction (57.1%). The least prevalent area of difficulty was infrequency (51.4%). The most prevalent area of severe difficulty in the females was non-communication (17.1%) followed by non-sensuality (15.7%). Unlike the males however, infrequency was the least prevalent area of severe difficulty among the females.

## Discussion

The prevalence of SD amongst subjects with PD was 64.4% in males and 65.7% in females indicating a higher prevalence in females, which is consistent with earlier reports by N Amidu, WK Owiredu, CK Gyasi-Sarpong, E Woode and L Quaye [13] that recorded higher prevalence (61.5%) among able-bodied females than males(59.2%).The SD prevalence of 64.4% recorded in male participants in this study is similar but lower than was reported by N Amidu, WK Owiredu, E Woode, O Addai-Mensah, KC Gyasi-Sarpong and A Alhassan [9] in a study in which a prevalence of 65.9% was recorded. In another study by N Amidu, WK Owiredu, E Woode, O Addai-Mensah, L Quaye, A Alhassan and EA Tagoe [10] an SD prevalence of 72.8% was recorded among female participants, thus confirming the generality of a higher prevalence amongst subjects without PD as compared to subjects with PD, thus similarities exists in the sexual functioning of people with and without PD.

The most affected areas of difficulty were infrequency, PE and non-sensuality in males whilst anorgasmia, non-communication and avoidance were the highest contributors to SD in females. Anorgasmia as an area of difficulty may be related to the prevalence of vaginismus in this study. In some cases, vaginismus impedes orgasm through blocking penetrative intercourse or by causing pain during thrusting. It is therefore not surprising, to observe a positive correlation between anorgasmia and vaginismus in this study. The contributions of infrequency amongst male participants could be due to unavailability of partners to engage in sexual activity either due to perceptions of available females to these subjects with PD or due to the restraint placed on them by societal perceptions which results in the lack of self-confidence which invariably affects their sexual expression.PE recorded as an area of difficulty among persons with PD could be due to spina bifida, multiple sclerosis, spinal injury, transverse myelitis or other forms of nervous damage which invariably leads to a poorer nervous control of sexual stimuli with resultant PE. This could explain why female subjects with PD recorded anorgasmia as the highest area of difficulty.

Male participants who consumed alcohol were about five times more likely to develop SD. Alcohol in small amounts is known to have a relaxant effect and subsequently improve sexual performance but regular and increased consumption is known to reduce genital response [23,24] and this poses a potential risk of causing SD. It is interesting to observe also that alcohol was a risk factor among male participants but not among female participants with PD. Earlier reports by Amidu *et al.* among study participants without PD reported alcohol as a risk factor for the development of SD in females [10]. Although the female participants in this study who consumed alcohol were much lower (7.1%) than in these earlier reports and the presence of PD which could make these subjects wary of alcohol consumption and could explain why the females in this study did not record alcohol consumption as a risk factor. Whatever could have resulted in the differences in these subjects, alcohol consumption has been reported by several authorities to have an effect on sexual function [10,25,26]. Sexual dysfunction was linked to a lower quality of life, for the disabled however, the issue may even be worse considering the negative views about sexual acts they tend to receive from society. Quality of life is a multidimensional construct, incorporating at least physical, psychological, and social well-being [27]. Sexuality and intimacy are considered central to a person’s well-being and are, as such, important aspects of quality of life of a person [28].

The average monthly income was recorded as GHC 160.00, with more than half of subjects earning less than GHC100 per month. Although income levels was not recorded in this study as a risk factor, the inability to earn enough income could be a barrier to full social integration that could largely affect the willingness and desire to engage in sexual relations. The lower educational standards in females could also play a major role in their low earnings as few of them will find occupation and jobs that will pay well. The societal myths and perceptions could also place a barrier on the willingness of employers to engage the services of people with PD thus further compounding their situation. People with PD have needs that are largely the same as their able-bodied counterparts and the fact that society has largely not been responsive to their welfare and full integration poses a barrier to their needs including their sexuality. The constellation of physical, psychological, and emotional challenges that persons with PD endure as a result of disability must be of concern to both healthcare providers as well as their families and thus their sexuality and its needs has to be addressed with a recognition of the importance of the recovery of emotional well-being and sexual function.

The observation of a higher SQoL amongst female subjects than males is not surprising since intimacy and sexual bonding continue to exist throughout marital life whereas the income level of this population is generally very low. Male study participants thus remain sexually active even if they earn very little but not without an impact on their sexual quality of life.

A poor sexual functioning coupled with a lower sexual satisfaction are risk factors for poor quality of life. Generally, the males in this study had significantly lower sexual quality of life than the females. Sexual concerns of patients suffering from neurological diseases are common [29] but still remain poorly understood in clinical practice [30].

MP McCabe and G Taleporos [31] in their assessment of SD in 1,196 subjects with physical impairment demonstrated that people who had more severe physical impairments had significantly lower levels of self-esteem and sexual satisfaction and were sexually active less frequently and are more depressed than able-bodied people. Until rehabilitation of the physically disabled is viewed from the holistic and comprehensive dimensions challenges to their quality of life will always exist and hinder their full integration into society.

## Conclusion

The prevalence of SD amongst persons with PD is 64.4% in males and 65.7% in females. Physically disabled men are more likely to consume alcohol than females and this increases their risk of developing SD by five-fold. Physically disabled women enjoy a better sexual quality of life than physically disabled men.
